# C_60_ as fine fillers to improve poly(phenylene sulfide) electrical conductivity and mechanical property

**DOI:** 10.1038/s41598-017-04491-1

**Published:** 2017-06-30

**Authors:** Maliang Zhang, Xiaotian Wang, Yali Bai, Zhenhuan Li, Bowen Cheng

**Affiliations:** State Key Laboratory of Separation Membranes and Membrane Processes, School of Materials Science and Engineering, Tianjin Polytechnic University, 300160 Tianjin, China

## Abstract

Electrical conductive poly(phenylene sulfide) (PPS)/fullerene (C_60_) composites were prepared by 1-chlornaphthalene blending method, and the interface effects of C_60_ and PPS on PPS/C_60_ properties were characterized. C_60_ is an excellent nanofiller for PPS, and 2 wt% PPS/C_60_ composite displayed the optimal conductivity which achieved 1.67 × 10^−2^ S/cm. However, when C_60_ concentration reached 2 wt%, the breaking strength and tensile modulus of PPS/C_60_ fiber achieved maximum 290 MPa and 605 MPa, and those values were 7.72 and 11.2 times as that of pure PPS. The excellent conductive and mechanical properties of PPS/C_60_ were attributed to the heterogeneous nucleation of C_60_ during PPS crystallization, formation of a large number of covalent bond by main C_60_-thiol adducts and minor C_60_-ArCl alkylation between C_60_ outer surface and PPS matrix. At same time, PPS/C_60_ thermal properties were also investigated.

## Introduction

Fullerene/polymer composites have recently attracted considerable attention from the materials research community^1–3^. It has been shown that the incorporating fullerene or its derivatives into a polymer matrix could significantly improve its photovoltaic, electromechanical and thermomechanical properties^4, 5^. The literature already identified several techniques to blend fullerene with polymers^3^, and the properties of fullerene/polymer composites are closely related to the morphologies of the polymers formed during melt or solution processing^3, 4^. Chemical functionalization of fullerenes is often performed to increase the miscibility of fullerenes with host polymers^6^, but the chemical functionalization method makes fullerenes lost certain original precious properties, which resulted in chemical attachment of fullerenes to a polymer is not always the most effective method for getting high performance composite materials, despite a number of fullerene-attached polymers have been successfully synthesized in the past^7^.

PPS is widely used in practice due to its excellent properties, such as chemical resistance, low coefficient of friction, mechanical behaviors, dimensional stability and electrical property^8–10^. To improve the performance of PPS, the nanofiller-reinforced composites require homogenous filler dispersion and good interfacial adhesion with the host matrix. Attaching functional groups onto the filler surface has been proven to be an effective approach to prepare polymer composites, and another effective method is wrapping fillers by organic molecules to provide the π-π stacking interactions between PPS matrix and nanofiller sidewalls. The conventional fillers included single-walled carbon nanotubes (SWCNTs)^11, 12^, inorganic fullerene-like tungsten disulfide (IF-WS_2_)^13^, SWCNT-IF-WS_2_^14–16^, functionalized SWCNTs and MWCNTs^17–20^, nano particles of TiO_2_, ZnO, CuO and SiC^21^, nanoscale alumina particles^22^, graphite^23^, glass fiber^24^, metal inorganic salt^25^, nano-SiOx^26^, carbon and fiber^27^ *et al*. However, it has been recently shown that the properties of PPS based composites hardly increase at low nanofiller loadings (up to 1 wt%)^14, 28, 29^. Because of van der Waals attraction between C_60_ and their large surface area, C_60_ tend to form agglomerates during mixing with PPS by melt blending. Therefore, it is difficult to use the conventional method to disperse C_60_ in the PPS matrix.

Herein, C_60_ was selected as fillers to improve PPS electrical conductivity and mechanical property, and the influence of the 1-chloronaphthalene solution mixing method and subsequent melt process on filler dispersion, C_60_ interfacial adhesion with PPS matrix and composite properties were investigated to correlate the microscopic structure with macroscopic properties.

## Experimental

### Materials and reagents

C_60_ powder (>99.9 wt/wt purity) was purchased from Puyang Yongxin Fullerene Co., Ltd, and used as received. PPS (Mw~3.3 × 10^4^, Mw/Mn~1.4 × 10^4^, d25 °C~1.35 g/cm^3^, Tg~90 °C, Tm~280 °C) was synthesized from Na_2_S and 1,4-dichlorobenzene in N-methylpyrrole, and the end groups of synthesized PPS contained –SH and -PhCl. 1-chlornaphthalene (95%, Fluka) was purchased from J& K Chemical Co., Ltd and purified by distillation under reduced pressure before use.

### Sample preparation

PPS (5 g) was dissolved in 100 ml of 1-chloronaphthalene at 205 °C under nitrogen atmosphere. After that, a certain amount of C_60_ ranging from 0.5 to 10 wt% were loaded into PPS/1-chloronaphthalene solution at 205 °C under mechanical agitation. PPS/C_60_ composites with 0.5 to 10 wt% nanofiller were obtained after removing 1-chloronaphthalene under vacuum condition. And then PPS and PPS/C_60_ composite fibers with diameters of 45~85 µm were prepared by melt spinning technology at 315 °C.

### Characterization and Measurement

Fourier transform infrared spectroscopy (FT-IR) spectra were obtained using Bruker IFS66 at room temperature. Thermo gravimetric analyzer (TGA) analysis was carried out on a NETZSCH STA 409 TG analyzer, and the rate of temperature increase was at 10 °C/min. X-ray diffraction (XRD) data were obtained using an Elmer PHI-5600 instrument using a Mg Kα line as a radiation source and a D8 discover. The morphology of composites was characterized by a field emission scanning electron microscopy (FESEM, Hitachi 4800S, and Japans) and transmission electron micrographs (TEM, Hitachi H-7650 microscope). Differential scanning calorimetry (DSC) analysis was performed on a Perkin Elemer DSC-7 under nitrogen condition, and samples placed in aluminium pans were melted at 320 °C and kept at this temperature for 5 min to erase their thermal history. Subsequently, they were cooled from the melt to room temperature and then heated again up to 320 °C at a scan rate of 10 °C/min. From the DSC heating and cooling traces, peak melting temperature (*T*_*m*_), heat of melting (Δ*H*_*m*_), peak crystallization temperature (${T}_{c}$) and heat of crystallize (Δ*H*_*c*_) were obtained. The degree of crystallinity (${X}_{c}$) was calculated from the following equation:

1 $${X}_{c}( \% )=\frac{{\rm{\Delta }}{H}_{c}}{{\rm{\Delta }}{H}_{f}(1-{W}_{f})}\times 100 \% $$

Where Δ*H*_*c*_ is the cold crystallization enthalpy from the DSC scan, ${W}_{f}$ is the weight fraction of C_60_ in composites, and Δ*H*_*f*_ is the melting enthalpy of 100% crystallized PPS which was taken as 105 J/g^23^.

Fiber diameter data were obtained using a KEYENCE VHX-1000 microscope at room temperature. The breaking strength (${\delta }_{t}$), breaking elongation ($\varepsilon$) and tensile modulus (MPa) were measured on single fiber strength tester (China LLY-06). Each sample was tested ten times to evaluate the average value. The breaking strength was calculated from the following equation:

2 $${\delta }_{t}(MPa)=4\frac{{F}_{b}}{\pi {d}^{2}}\times {10}^{-6}$$

Where *F*_*b*_ is the maximum tension value, *d* is the fiber diameter. The breaking elongation was determined by the following equation:

3 $$\varepsilon ( \% )=\frac{L-{L}_{0}}{{L}_{0}}$$

Where *ε* is the breaking elongation, *L* is the length of fiber elongation, *L*_0_ is the initial length of the fiber before test. The fiber tensile modulus (*E* MPa) was determined by the following equation:

4 $$E=\frac{{\delta }_{t}}{\varepsilon }=\frac{4{F}_{b}{L}_{0}}{\pi {d}^{2}(L-{L}_{0})}\times {10}^{-4}$$

Electrical conductivity of samples was measured by the four-point probe method using a Scientific Equipment device with a spacing probe S = 0.2 cm equipped with a DC precision power source (Model LCS-02) and a digital microvoltmeter (Model DMV-001). The powder sample was filled into a test slot, then applying a pressure about 18 MPa. The electrical resistivity was calculated from the following equation:

5 $${\rm{Electrical}}\,{\rm{resistivity}}={\rm{2}}\,{\rm{\pi }}S\times (W/S)\times D\times (V/I)$$

Where S is the distance of adjacent probe, W is the sample thickness, D is the position correction factor, V is the test voltage, I is the test current. Each sample has been tested ten times, and the electrical resistivity is the average value of 10 measurement. The electrical conductivity of samples was calculated through the following equations:

6 $${\rm{Electrical}}\,{\rm{conductivity}}=1/\mathrm{Electrical}\,{\rm{resistivity}}$$

## Results

**Figure 1:**
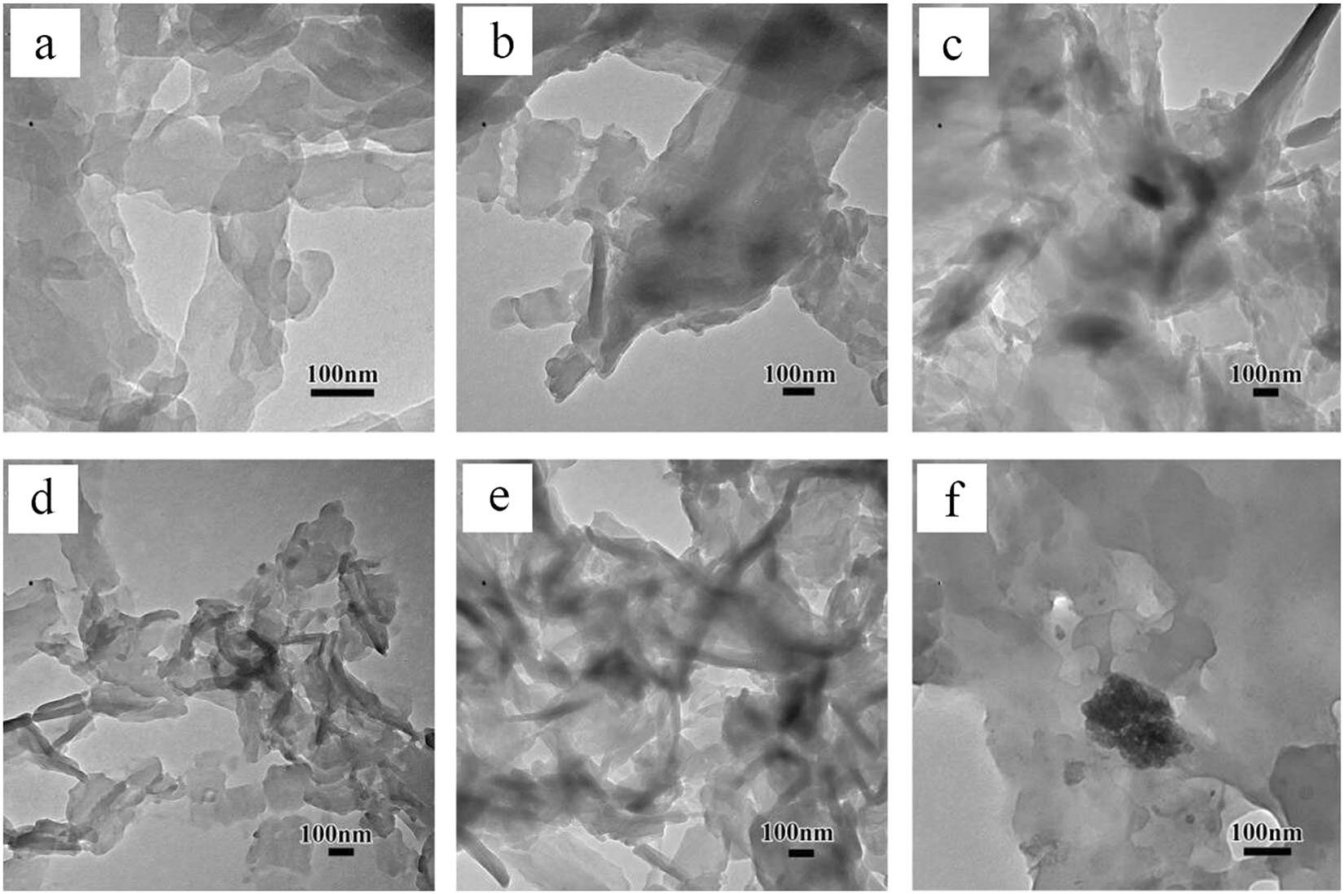
TEM micrographs of PPS/C_60_ powder with different weight fractions of C_60_ (**a**) Pure PPS; (**b**) 0.5 wt%; (**c**) 1 wt%; (**d**) 2 wt%; (**e**) 5 wt%; (**f**) 10 wt%.

After C_60_ and PPS were dissolved in 1-chloronaphthalene, 1-chlornaphthalene evaporated under vacuum condition to obtain PPS/C_60_ composites, and the dispersion and alignment of C_60_ within the PPS matrix were characterized by TEM (Fig. 1). The dark and light areas correspond to nanofillers and PPS matix, respectively. The image of C_60_ showed a homogeneous size distribution with average diameter around 300 nm, which revealed C_60_ was easily agglomerated due to the strong van der Waals forces and intensive π-π stacking interactions among C_60_. However, the micrograph of 0.5–2 wt% C_60_/PPS composites indicated the aggregate C_60_ diameter decrease, and no voids or discontinuities are detected between the C_60_ outer surface and PPS matrix. The TEM micrograph of 0.5–2 wt% PPS/C_60_ fibers indicated that C_60_ was well dispersed into PPS matrix, however, when much more C_60_ was incorporated into PPS (such as 10 wt% PPS/C_60_), C_60_ formed agglomerates inside the PPS matrix. TEM observation showed that C_60_ were wrapped in PPS or covered by PPS layer and the heterogeneous dispersed bright dots with dimensions from 150–350 nm in 5 wt% and 10 wt% PPS/C60 composites were detected, indicating good adhesion between C_60_ and PPS.

**Figure 2:**
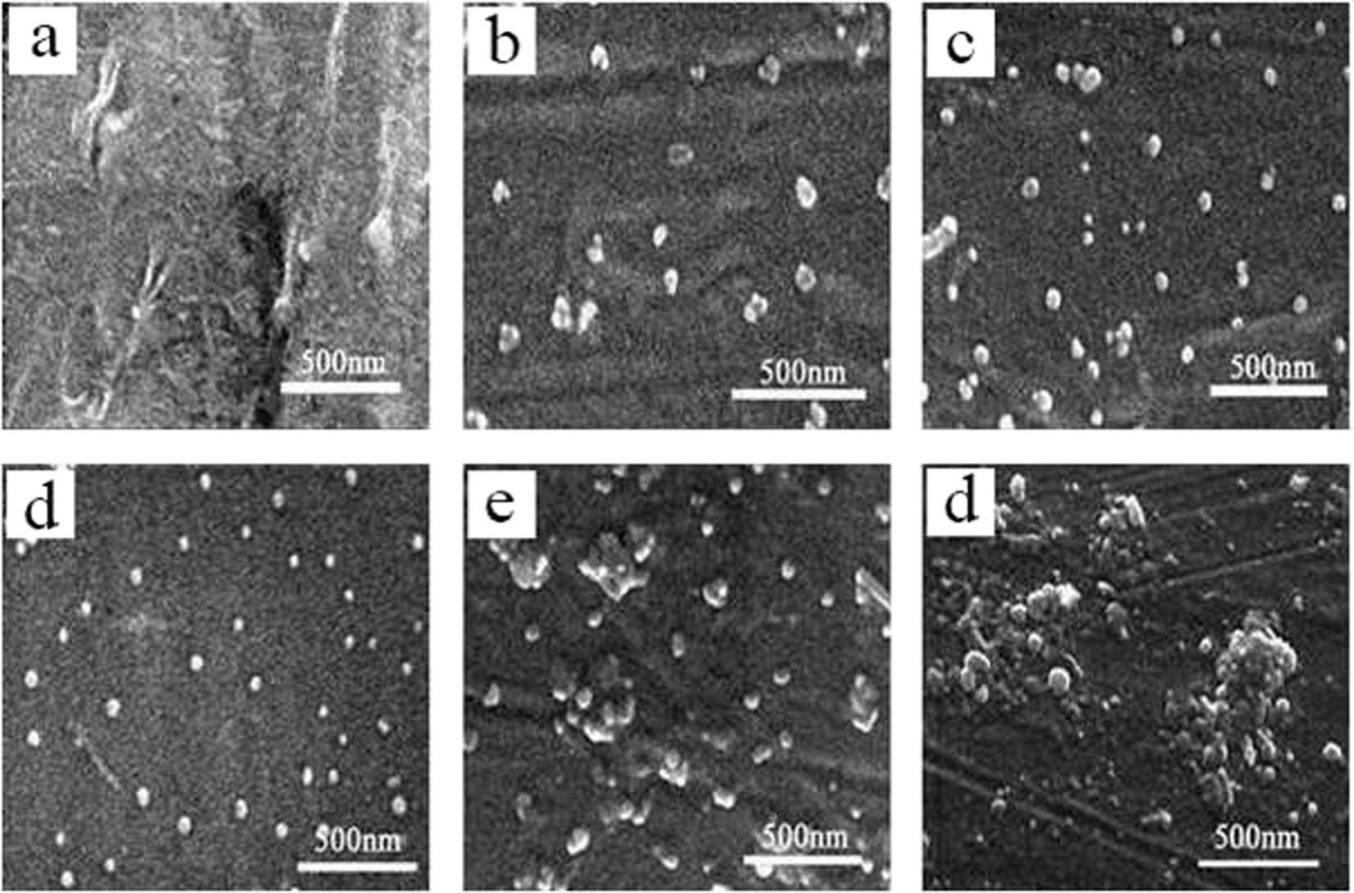
SEM micrographs of cryogenically fractured melt-processed PPS/C60 with different weight fractions of C_60_ (**a**) PPS; (**b**) 0.5 wt%; (**c**) 1 wt%; (**d**) 2 wt%; (**e**) 5 wt%; (**f**) 10 wt%.

**Figure 3:**
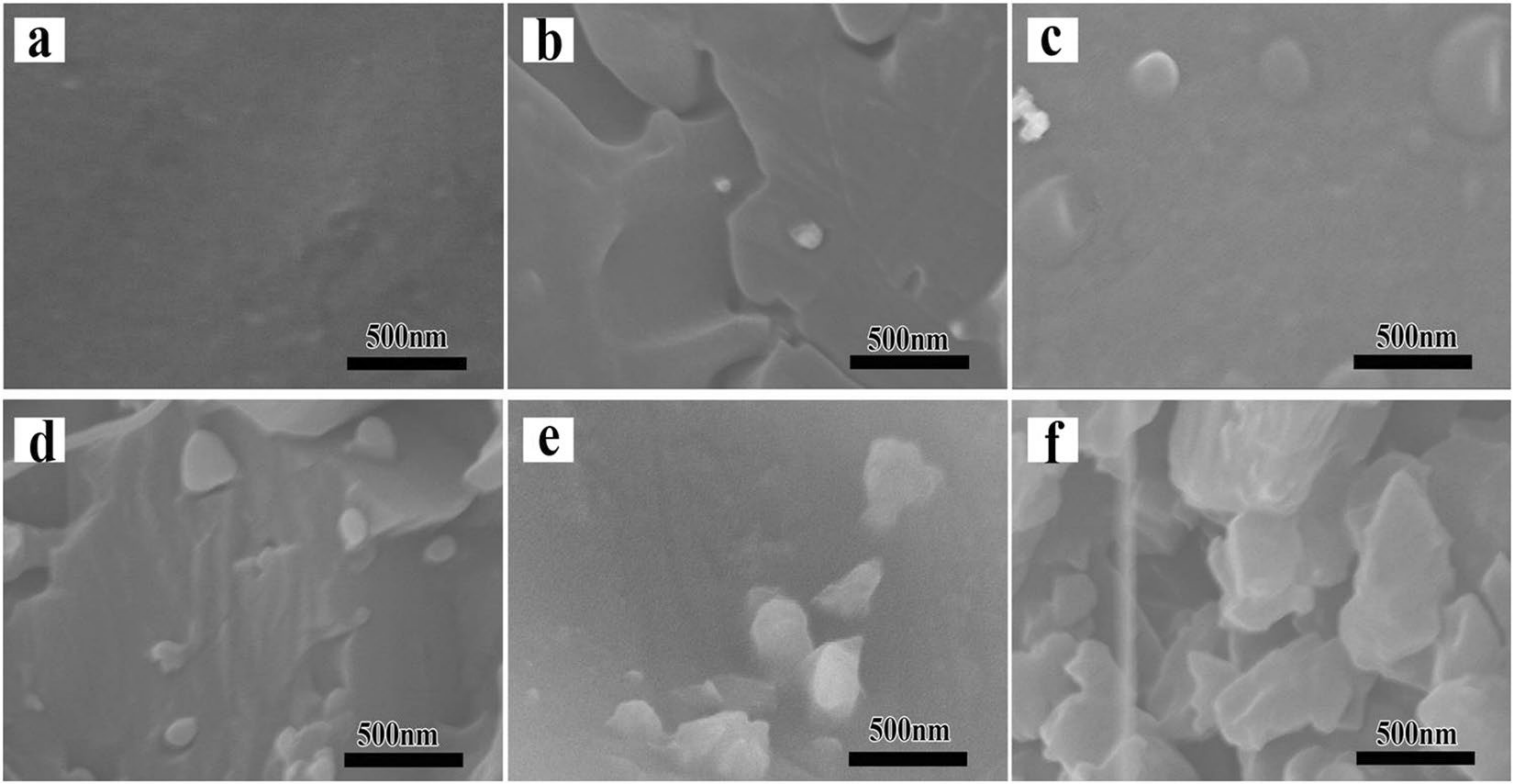
Cross-sectional SEM micrographs of cryogenically fractured melt-processed PPS/C_60_ with different weight fractions of C_60_ (**a**) PPS; (**b**) 0.5 wt%; (**c**) 1 wt%; (**d**) 2 wt%; (**e**) 5 wt%; (**f**) 10 wt%.

Figure 2 A shows the SEM micrograph of PPS as a blank example, and the SEM images of Fig. 2B,C,D indicated that almost no apparent C_60_ agglomerates were detected in 0.5 wt%, 1 wt% and 2 wt% PPS/C_60_ composites, due to C_60_ nanofillers further disaggregation within the polymer matrix during PPS/C_60_ melt-process. C_60_ nanofillers were randomly dispersed in the PPS matrix, occurring irregular crystal with dimensions from 20–50 nm when the content of C_60_ is less than 2 wt%. C_60_ nanofillers are difficult to disperse uniformly in PPS melt, therefore PPS can not well reinforced by C_60_ nanofillers in melt blending technology. However, C_60_ aggregates can be well dispersed in PPS matrix by solution blending method, in which the solvent plays a dual role in dispersing C_60_ and preventing C_60_ agglomeration. The heterogeneous dispersed bright dots with dimensions from 80–350 nm in 5 wt% and 10 wt% PPS/C_60_ composites were detected, which was attributed to C_60_ agglomerates. Similar results can also be found through the cross-sectional SEM images of the composites in Fig. 3. For PPS/C_60_, 2 wt% C_60_ particles can be uniformly dispersed in PPS matrix, but further addition of 5 wt% C_60_ would cause the agglomeration of C_60_, as can be seen in Fig. 3e and f.

**Figure 4:**
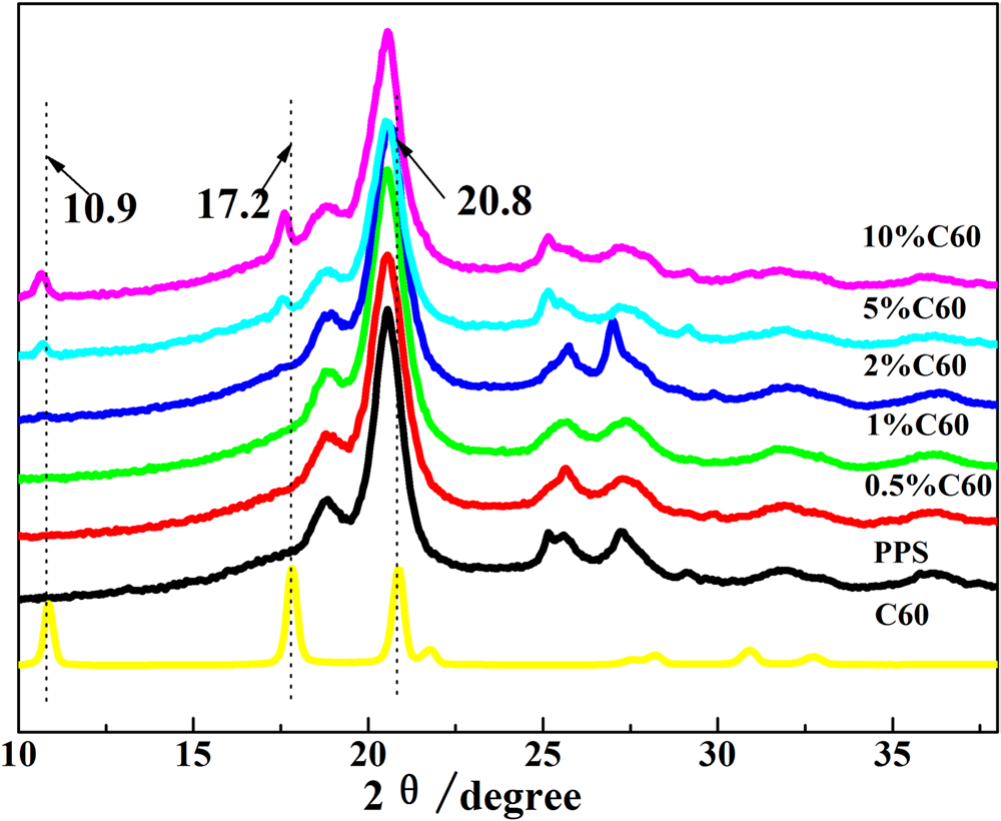
XRD spectra of C_60_, PPS and PPS/C_60_ composite powder.

X-ray diffraction patterns of PPS/C_60_ composites were displayed in Fig. 4. The two diffraction peaks at 19.2° and 20.8° are corresponding to the (110) and (200) crystalline planes of the orthorhombic structure of PPS^30^. And C_60_ shows characteristic peaks at 2θ = 10.9°, 17.2°, 20.8°, 21.8°, 28.2°, 30.8° and 32.7° arising from the (110), (220), (310), (220), (330), (420) and (330) crystalline planes of the orthorhombic unit cell, respectively^31^. The C_60_ agglomerate peaks were hardly visible in the diffractograms of 0.5–2wt% composites, which suggested that C_60_ was well dispersed in PPS matrix. After 5 wt% C_60_ was introduced into PPS, the serious C_60_ agglomerate was clearly observed, and the crystal characteristic peaks of C_60_ shifted to lower 2θ values bacause C_60_ aggregates result in PPS lattice distortion^32^. Moreover, composite peaks become broader with reduced intensity, indicating the structural order decline which induced by the incorporation of C_60_. These observations are consistent with the behaviors of SWCNTs^33^ and MWCNTs^34^, where the local order of the polymer decreased after the grafting reaction.

**Figure 5:**
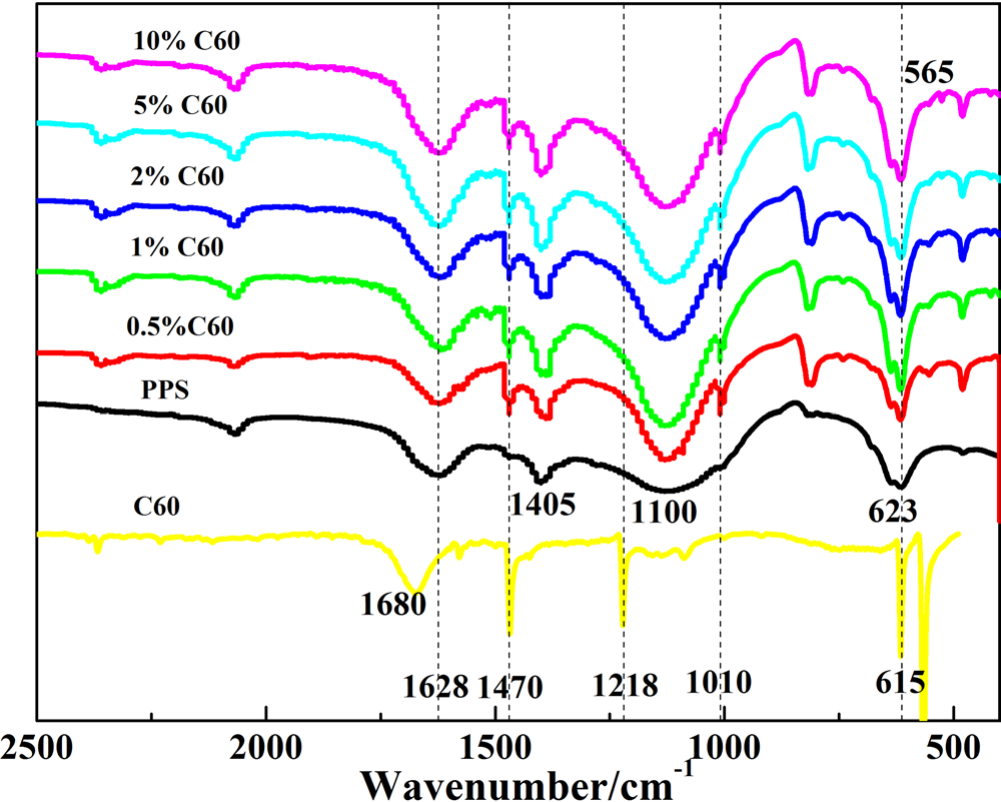
FT-IR spectra of C_60_, PPS and PPS/C_60_ composite powder.

**Figure 6:**
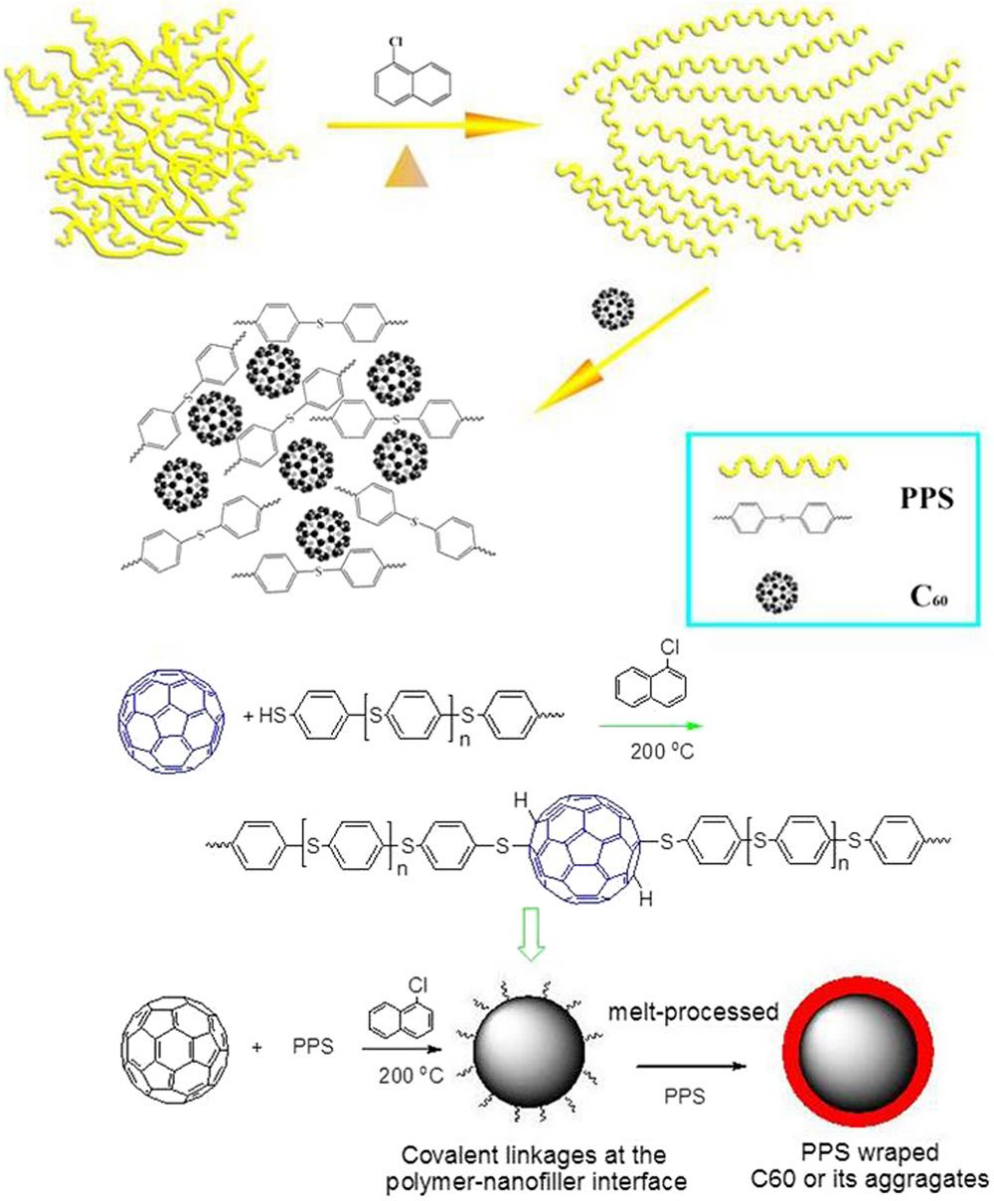
The chemical functionalization process of fullerenes and PPS/C_60_ interfacial adhesion changes during solution and melt processing.

As shown in Fig. 5, the main bands at 565, 615, 1218, 1470 and 1680 cm^−1^ are characteristic absorption peak of C_60_^35^. The phenyl groups of PPS exhibited the absorption peaks at 1628 and 1405 cm^−1^, and the two bands at 1100 and 623 cm^−1^ were attributed to aromatic C-S stretching vibrations. After PPS was reinforced by C_60_, C_60_ characteristic absorption peaks at 565 and 1475 cm^−1^ band were still observed, which demonstrated the C_60_ successful incorporation into PPS matrix. However, C_60_ band at 1218 cm^−1^ disappeared and a new peak at 1010 cm^−1^ appeared, which implied the well C_60_ dispersion and C_60_-S formation (Fig. 6)^36, 37^. Solvent could help the tangled PPS molecular chain creeping and stretching in 1-chloronaphthalene, which effectively contributes to the well dispersion of C_60_ in PPS matrix. Although the alkylation or acylation was an effective functionalization method for fullerene^38, 39^, herein a C_60_-thiol adducts by reacting C_60_ with the end group SH of PPS was easier to take place^32^, and the covalent bond formation improved the C_60_-matrix interfacial adhesion^6, 7^. It is general known that the formation of π-π stacking interactions can be characterized through the shift of –CH bond. It is clear that the C-H bond absorption peaks of PPS located at 808.7 and 818.8 cm^−1^. In PPS/C_60_ material, C-H vibration peak of PPS/C60 remain unchanged, which indicated that there only existed a weak π-π stacking interactions between C_60_ and PPS matrix^36, 40^.

In order to testify the existence of chain-extension reaction through the end-group reaction between PPS and C_60_, PPS and PPS/C_60_ molecular weight were characterized by HTGPC (Table 1). As C_60_ content increased from 0.5 wt% to 2 wt%, PPS/C_60_ composite molecular weight propagated from 3.30 × 10^4^ g·mol^−1^ to 4.19 × 10^4^ g·mol^−1^, and polydispersity index (PDI) increased from 2.22 to 2.56. Only some PPS chains could interact with C_60_ to lead to the longer polymer chains. However, when C_60_ content reached 5 wt%, 5 wt% PPS/C_60_ composite molecular weight declined to 3.71 × 10^4^ g·mol^−1^. The deviation could be attributed to much more the low molecular weight C_60_ loading. A similar result was previously reported by Peng, K. J. & Liu, Y. L.^41^. HTGPC characterized results suggested some covalent bond formation between PPS and C_60_ through the C_60_-thiol adducts and C_60_-ArCl alkylation.

**Table 1:** Molecular weight and polydispersity index of different PPS.

Composites (wt%)	Mw (10^4^ g·mol^−1^)	Mn (10^4^ g·mol^−1^)	PDI
PPS	3.30	1.49	2.22
0.5	3.42	1.45	2.36
1	3.70	1.53	2.42
2	4.19	1.63	2.56
5	3.71	1.37	2.69
10	2.80	0.88	3.19

**Figure 7:**
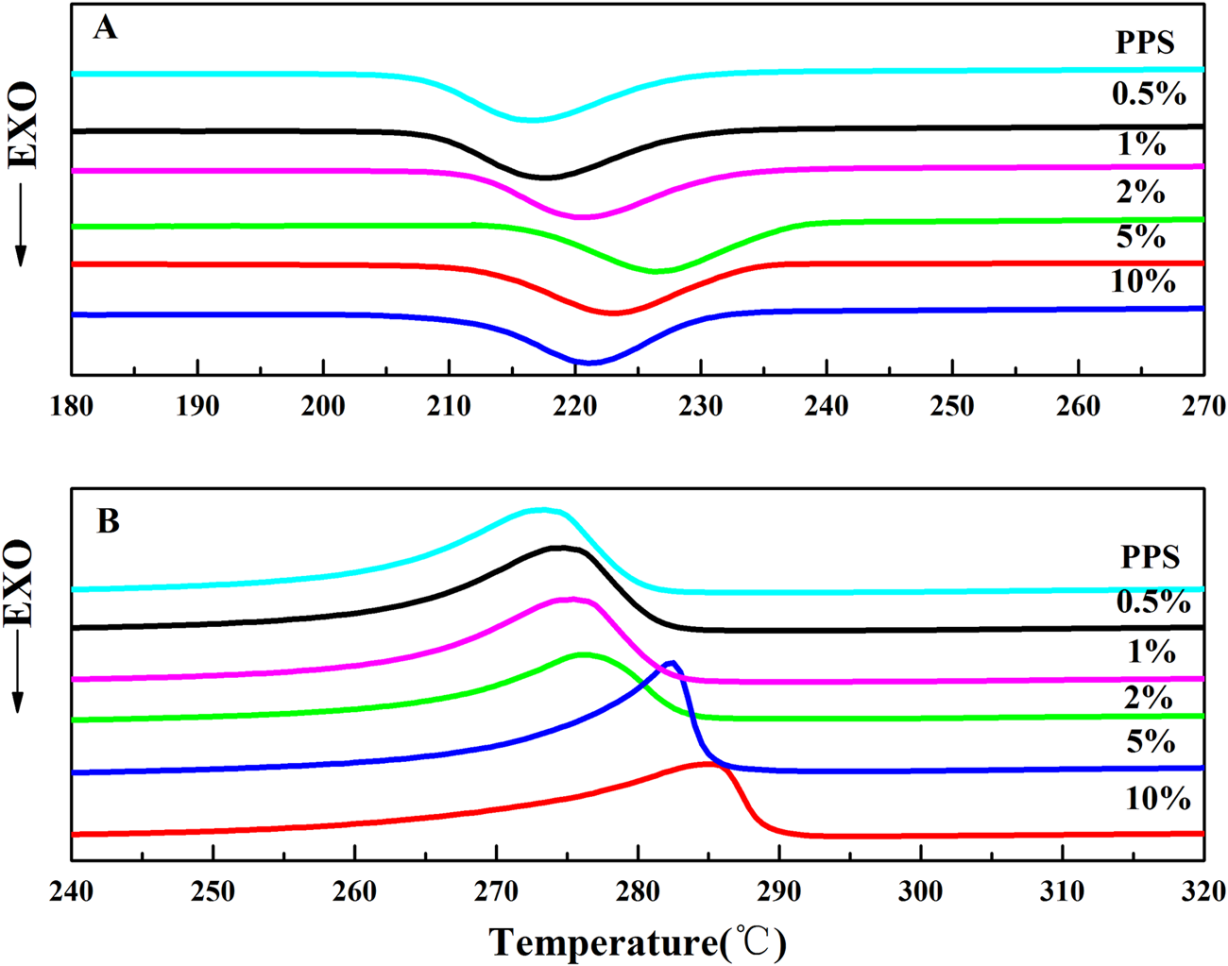
DSC thermograms obtained at a rate of 10 °C/min: (**a**) DSC cooling curves of PPS/C_60_; (**b**) DSC heating curves of PPS/C_60_.

The crystallization and melting behavior of composites were investigated (Fig. 7), and the calorimetric parameters derived from non-isothermal DSC scans were listed in Table 2. As it can be observed, 0.5 wt% C_60_ had less influence on PPS crystallization temperature (Tc) increase (Fig. 7a), and 0.5 wt% PPS/C_60_ exhibited *T*_*c*_ of 217.2 °C with Δ*H*_*c*_ being 42.98 J/g. However, for 2 wt% PPS/C_60_, *T*_*c*_ shifted to 227.6 °C with maximum Δ*H*_*c*_ being 48.23 J/g. AS for 5 wt% PPS/C_60_, *T*_*c*_ increases to 223.8 °C with Δ*H*_*c*_ decline to 42.42 J/g. The further increase of C_60_ concentration in composites slowed the mobility and diffusion of PPS chains, which led to a significant decline of PPS crystallization temperature. Those behaviors observed are in agreement with that reported by Jeon *et al*.^42^. It was worthy of noting that 0.5 wt% PPS/C_60_ could not well act as heterogeneous nucleating agent to accelerate PPS nucleation, which suggested that the intense restrictions on chain mobility are imposed by the C_60_–polymer chemical interactions.

**Table 2:** DSC traces for PPS and PPS/C_60_.

C_60_ content (wt%)	Tc (°C)	Δ*H*_*c*_ (J/g)	Tm (°C)	Δ*H*_*m*_ (J/g)	Xc (%)	Δ*T* (°C)
0	215.6	−49.11	272.5	43.43	46.77	38.7
0.5	217.2	−42.98	273.6	41.50	41.14	57.9
1	220.5	−44.22	275.6	42.10	42.54	55.6
2	227.6	−48.23	276.2	44.10	46.87	49.7
5	223.8	−42.42	282.4	40.14	44.89	61.7
10	221.7	−41.72	285.5	41.45	41.83	60.7

*T*_*m*_ shifted gradually to higher temperature with increasing C_60_ content (See Fig. 7b and Table 2). 0.5 wt% PPS/C_60_ exhibited *T*_*m*_ of 275.1 °C with Δ*Hm* being 41.50 J/g. As for 2 wt% PPS/C_60_, *T*_*m*_ shifted to 277.3 °C with maximum Δ*Hm* being 44.10 J/g. While for 5 wt% PPS/C_60_, *T*_*m*_ increased to 282.4 °C with Δ*Hm* decline to 40.14 J/g. In the case of 2 wt% PPS/C_60_, both Δ*H*_*m*_ and Δ*H*_*c*_ achieve the maximum, and *X*_*c*_ reaches the maximum value 46.87%.

**Figure 8:**
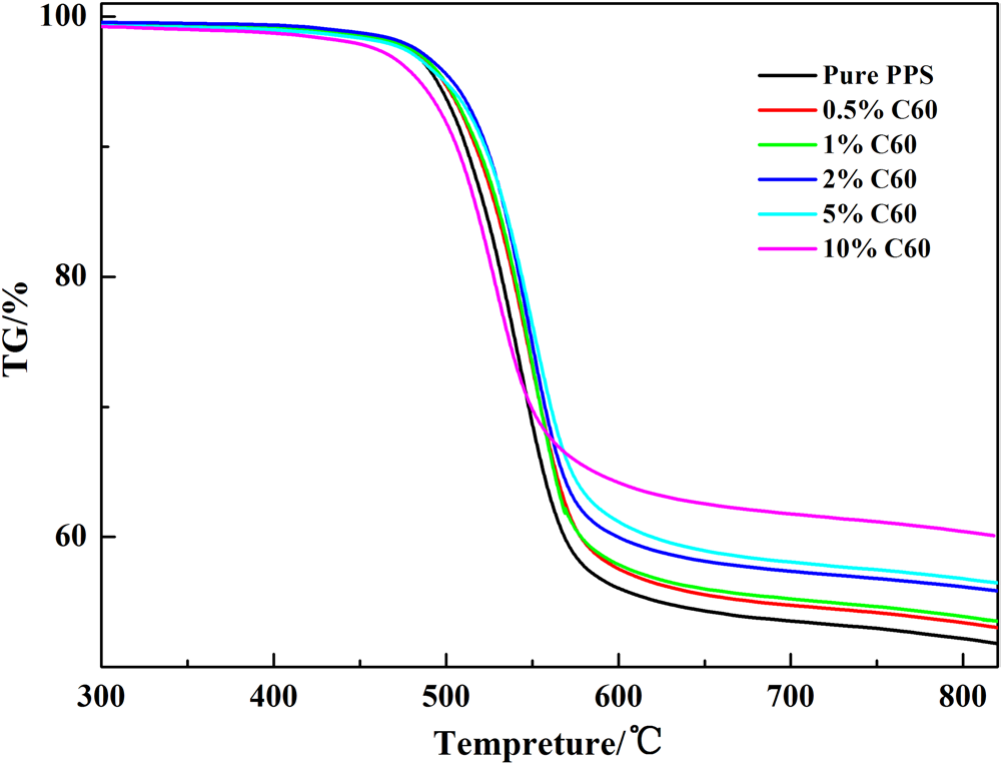
TG curves of PPS and PPS/C_60_).

The TG curves for the pure PPS matrix and composites under inert atmospheres were shown in Fig. 8, and their characteristic degradation parameters were summarized in Table 3. PPS displayed a single degradation stage that starts (T_i_) at 502 °C and exhibited the maximum weight loss (T_max_) rate at 541.3 °C. At 800 °C, the residual mass was about 52.2% of the initial weight. Clearly, the addition of 0.5–2 wt% C_60_ filler led to an improvement in the thermal stability of PPS matrix, and a maximum T_i_ increase (about 7 °C) was obtained at 2.0 wt% filler loading, and T_max_ increment for 2.0 wt% PPS/C_60_ was 5.5 °C. However, the significantly decline of T_i_ and T_max_ was detected at 10 wt% C_60_ loading, but the residual mass at 800 °C increased to 60.39% of the initial weight. Such results should be attributed to different factors. Firstly, C_60_ fillers are better dispersed within PPS matrix, which restricts chain mobility or diffusion to slow down the decomposition process^11^. Secondly, the covalent anchoring of PPS to C_60_ leads to a strong enhancement in the thermal conductivity that facilitates heat dissipation within the composite^43^. The reason for the thermal stability decline at 5 and 10 wt% C_60_ loading might be attributed to the appearance of C_60_ agglomerates.

**Figure 9:**
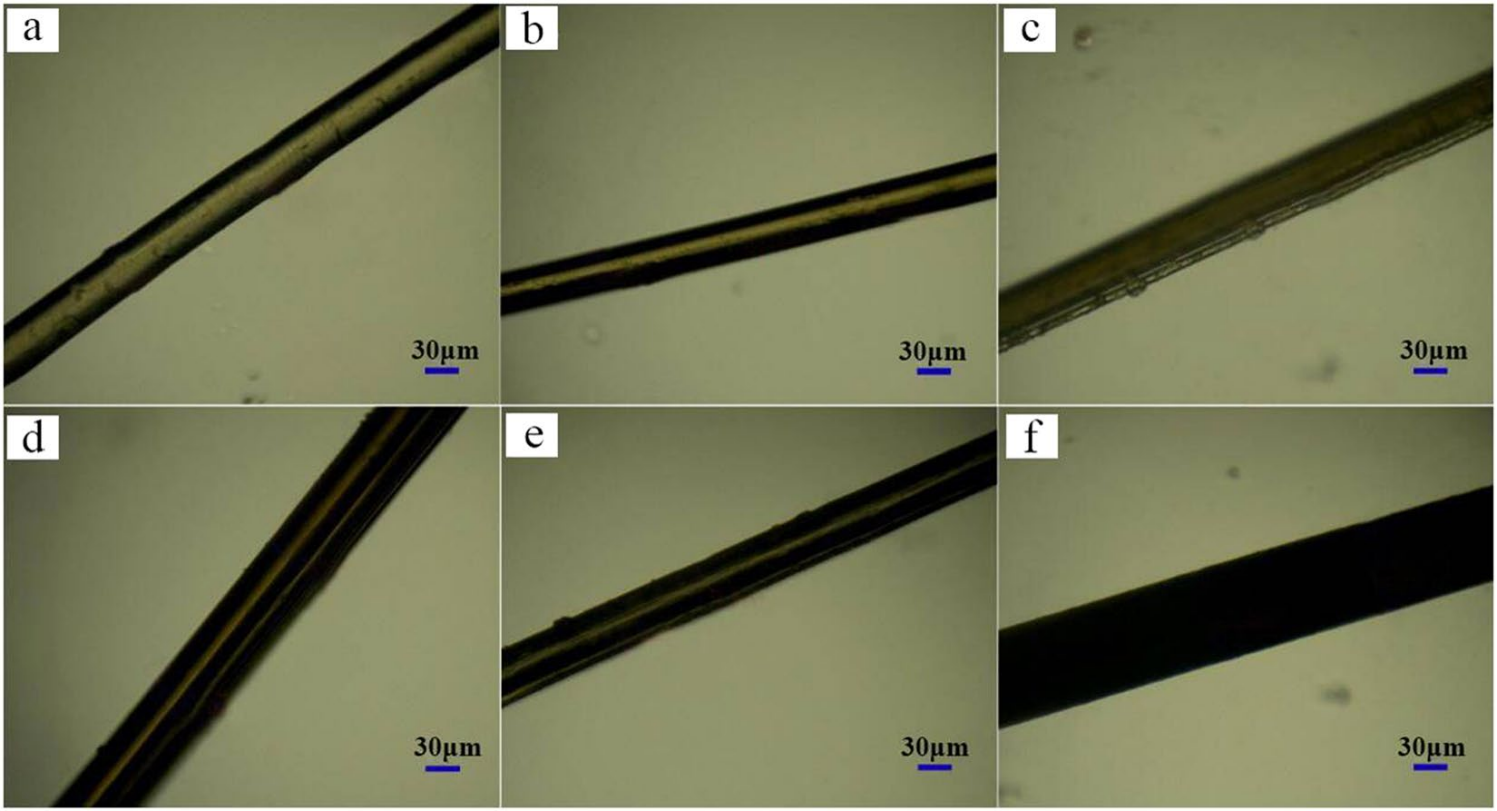
Micrographs of PPS/C_60_ with different weight fractions of C_60_ (**a**) PPS; (**b**) 0.5 wt%; (**c**) 1 wt%; (**d**) 2 wt%; (**e**) 5 wt%; (**f**) 10 wt%.

**Figure 10:**
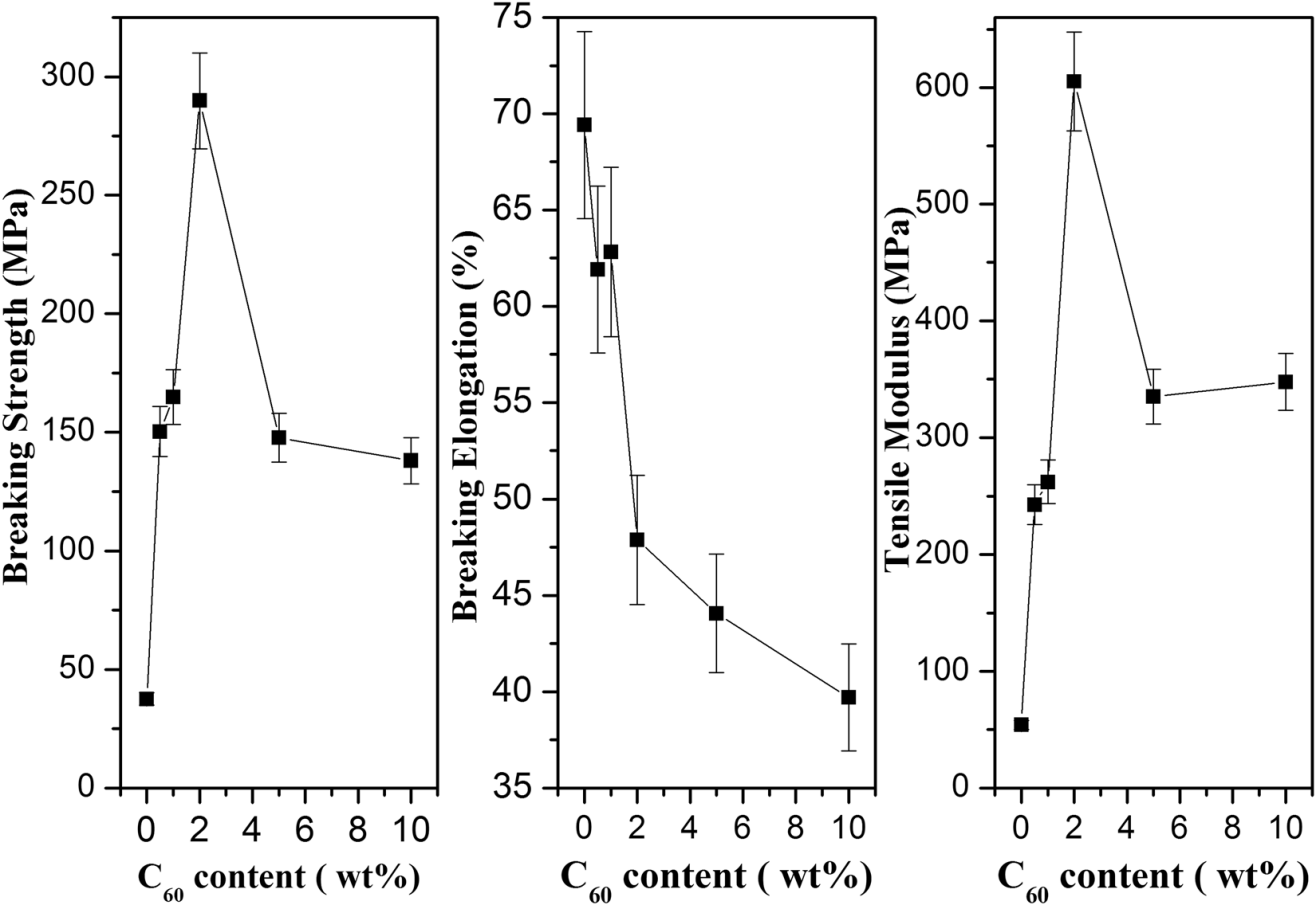
Mechanical properties of PPS/C_60_ fiber with different addition amount of C_60_: (**a**) breaking strength; (**b**) breaking elongation; (**c**) tensile modulus.

Figure 9 shows single fiber morphology of each sample respectively. The characterized results suggested the diameter of fibers were 45.2 ± 0.8 μm, 40.5 ± 0.5 μm, 42.5 ± 1.0 μm, 64.3 ± 1.0 μm, 47.6 ± 1.0 μm and 87.2 ± 0.8 μm, respectively, changing with the content of C_60_. The mechanical behavior of PPS/C_60_ fibers was investigated by single fiber strength tester technique which provides additional information about filler-matrix and filler-filler interactions. Figure 10 showed the breaking strength, breaking elongation and tensile modulus of PPS/C_60_ fiber. The results of mechanical property study indicated that the concentration of C_60_ had a greater influence on the mechanical performance of composites. As C_60_ nanofiller content increase, the breaking strength and tensile modulus of composites firstly increased and then decreased. When C_60_ concentration reached 2 wt%, the breaking strength and tensile modulus of composites achieved maximum 290 MPa and 605 MPa, and those value were 7.72 and 11.2 times as that of pure PPS, respectively. The breaking elongation of PPS/C_60_ composites always decreased with increasing C_60_ content (Fig. 10b). The excellent mechanical properties of PPS/C_60_ were attributed to the heterogeneous nucleation of C_60_ during PPS crystallization, the formation of a large number of covalent bond by C_60_-thiol adducts and π-π stacking interactions between C_60_ surface and PPS matrix. However, the excessive addition of C_60_ caused a significant reduction of breaking strength, e.g., the breaking strength of 10 wt% C_60_ composite declined to 148 MPa. The excessive addition of C_60_ reduced PPS crystallization degree and decreased the combination between PPS and C_60_, which were attributed to the phenomenon of C_60_ aggregation. These results can also confirm by the SEM and TEM images of PPS/C_60_ composites presented in Figs 1–2.

**Figure 11:**
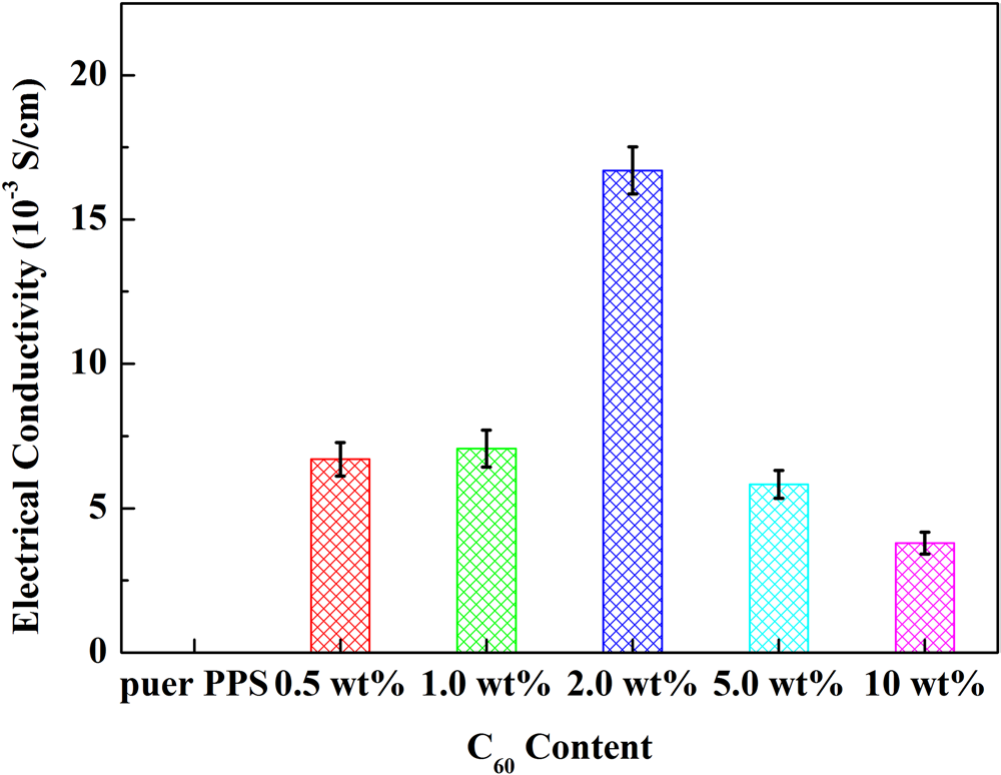
Room temperature electrical conductivity (r) of PPS/_C60_ composites.

PPS is an insulating material (σ 10^−16^ S/cm), which limits its use in self-health monitoring, electro-actuation, etc^32^. Herein, C_60_ was used as conductive fillers to improve PPS electrical conductivity and the electrical performance of PPS/C_60_ composites were compared (Fig. 11). As C_60_ nanofiller content increase, the electrical conductivity of PPS/C_60_ composites firstly increased and then decreased. When C_60_ concentration reached 2 wt%, the electrical conductivity of composites achieved maximum 1.67 × 10^−2^S/cm, much higher than the value of pure PPS. The excellent electrical conductivity of 2 wt% PPS/C_60_ composites was attributed to the well dispersed C_60_ fillers, and the maximum conductive networks might be formed in the composite at appropriate C_60_ content because of the conductive network formation^44^. C_60_ fillers are well dispersed in the PPS matrix owing to the covalent bond formation by main C_60_-thiol adducts and mino C_60_-ArCl alkylation between C_60_ surface and PPS. However, the excessive addition of C_60_ nanofillers caused a significant reduction of electrical conductivity, e.g., the electrical conductivity of 10 wt% C_60_ composite declined to 3.79 × 10^−3^S/cm, due to the phenomenon of C_60_ aggregation.

## Conclusions

PPS composites with well-dispersed C_60_ had been prepared by solution co-blending method because solvent promotes the tangled PPS molecular chains creeping and stretching. The electrical conductivity value of 2 wt% achieved the maximum, and the excellent electrical conductivity of 2 wt% PPS/C_60_ composites was mainly attributed to the covalent bond formation by main C_60_-thiol adducts and minor C_60_-ArCl alkylation between C_60_ surface and PPS, but C_60_ aggregation reduced composite electrical conductivity. Furthermore, 2 wt% C_60_ could effectively increase PPS crystalli zation temperature, thermal stability and mechanical performance. However, the excessive C_60_ loading reduced the PPS crystallization degree and caused C_60_ re-aggregation, which led to poorer mechanical performance of PPS/C_60_ composite.

**Table 3:** Thermogravimetric data under nitrogen atmosphere.

C_60_ content (wt%)	T_i_ (°C)	T_max_ (°C)	The residual mass at 800 °C (wt%)
0	502.0	541.3	52.16
0.5	506.4	546.3	52.37
1	507.2	546.6	53.78
2	509.0	546.8	56.21
5	505.5	544.0	56.79
10	497.6	534.0	60.39
